# Biomimetic Core–Sheath GelMA/PCL Nanofibers for Enhanced Peripheral Nerve Regeneration

**DOI:** 10.3390/polym18101241

**Published:** 2026-05-19

**Authors:** Xingxing Fang, Haichang Guo, Fei Yu, Wei Zhang, Qicheng Li, Shulin Bai, Peixun Zhang

**Affiliations:** 1 Department of Spine Surgery, The Third Affiliated Hospital of Sun Yat-sen University, Guangzhou 510630, China; fangxx5@mail.sysu.edu.cn (X.F.);; 2 Department of Electronic Engineering, The Chinese University of Hong Kong, Hong Kong 999077, China; 3 Department of Spine Surgery, Shenzhen Second People’s Hospital, The First Affiliated Hospital of Shenzhen University, Shenzhen 518035, China; 4 Department of Orthopedics and Trauma, Peking University People’s Hospital, Beijing 100044, China; 5 School of Materials Science and Engineering, Peking University, Beijing 100871, China

**Keywords:** GelMA, core–sheath, nanofibers, phase separation, peripheral nerve regeneration

## Abstract

Artificial nerve guidance conduits (NGCs) have gained significant attention in the field of peripheral nerve regeneration for the treatment of critically sized nerve defects. Nanotechnology-based NGCs are being explored as potential solutions for repairing and reconstructing peripheral nerve injuries due to their unique structure and topography. In this study, we present a novel core–sheath GelMA/PCL nanofiber construct fabricated through electrospinning and phase separation methods. The core–sheath GelMA/PCL nanofibers replicate the topological morphology of the native extracellular matrix (ECM). The outer layer, composed of GelMA, serves as an “adhesion domain” facilitating direct interaction with surrounding cells and tissues while improving wettability, integrin-mediated cell adhesion/attachment, and degradation. PCL, acting as the “elastic domain” within the nanofibers, enhances mechanical properties, maintains long-term stability of the NGCs, and enables controlled release of GelMA. Histomorphometric analysis along with electrophysiological and behavioral assessments demonstrate that these core–sheath GelMA/PCL nanofiber-based NGCs can activate endogenous mechanisms for peripheral nerve repair while promoting sensory/motor nerve regeneration and functional recovery. Overall, our findings demonstrate that GelMA/PCL nanofibers within the nuclear sheath can effectively remodel the nerve regeneration microenvironment by integrating “mechanical- biochemical” signals, thereby offering a novel strategy for addressing critical-size nerve defects.

## 1. Introduction

Peripheral nerve injuries (PNIs) caused by trauma, cancer, or congenital defects are recognized as significant clinical challenges [1,2,3]. Annually, approximately 100,000 individuals in the United States and Europe undergo peripheral nerve surgeries [4], resulting in healthcare expenses of around $150 billion in the US alone [5]. These patients often experience motor and/or sensory dysfunction due to the lack of effective and reliable repair techniques.

While autologous nerve grafts are considered the clinical gold standard for nerve repair, they are associated with limitations such as limited donor availability, donor site morbidity, the need for secondary surgery, immunological complications, and potential mismatch issues [6]. Consequently, a wide range of artificial nerve grafts composed of both natural and synthetic biomaterials have been investigated to address these neurological deficits [7,8].

Gelatin methacryloyl (GelMA) is a natural biomaterial characterized by its excellent bifunctionality and tunable physical properties, containing numerous amino acid groups [9]. Previous studies have demonstrated that GelMA-based hybrid materials can effectively promote the growth, elongation, and gene expression of nerve-associated cells [10,11]. However, the inherent limitations of GelMA, including its low mechanical strength and poor long-term stability (degrading within 2 weeks post-implantation), restrict its in vivo application in peripheral nerve tissue engineering. Polycaprolactone (PCL), a synthetic biomaterial with superior mechanical properties and appropriate biodegradability, has emerged as an ideal candidate for reinforcing hybrid scaffolds. It provides enhanced mechanical support and long-term stability, thereby facilitating the regeneration of nerves within the conduit [12,13].

**Figure 1:**
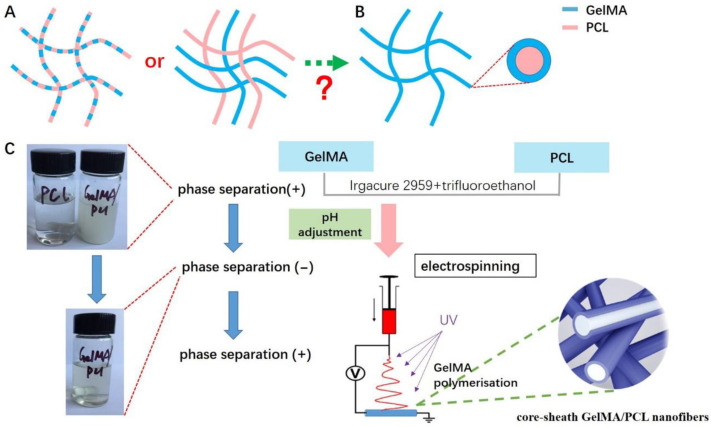
(**A**) Schematic illustration of the cross-linked nanofiber scaffolds composed of GelMA and PCL; (**B**) Schematic illustration of the core–sheath GelMA/PCL nanofibers; (**C**) Schematic illustration of the electrospinning process and the production process of core–sheath GelMA/PCL nanofibers. (The label on the left bottle reads “PCL”, the label on the right bottle reads “GelMA/PCL”, and the label on the bottom bottle also reads “GelMA/PCL”).

The hydrogen bonds between gelatin molecules (amino acid groups) are disrupted in a trifluoroethanol solution, leading to a linear structure of the gelatin molecules. This occurs because the pH of the solution is far from the isoelectric point, resulting in a transparent GelMA solution. The addition of the GelMA–trifluoroethanol solution to PCL (a hydrophobic substance) disrupts the balance, causing GelMA to aggregate and precipitate (Figure 1C, top left corner). This phenomenon occurs as the hydrophilic groups of GelMA orient inward while the hydrophobic groups expose outward. Previous studies have reported that mixing GelMA with PCL followed by electrospinning can produce cross-linked nanofiber scaffolds composed of both materials (Figure 1A) [14,15]. However, due to the degradability of GelMA, these GelMA-PCL scaffolds tend to collapse over time, which does not meet the long-term structural stability requirements for nerve conduits. To address this issue, researchers have incorporated other substances into GelMA/PCL composites, such as graphene and its derivatives [16,17], polypyrrole [18], and beta-tricalcium phosphate [19]. These additives result in nanofibers with an interleaved structure of GelMA and PCL. A recent study has demonstrated the preparation of GelMA-coated PCL fibers using coaxial electrospinning [14]. Nevertheless, the problem of precipitation caused by phase separation when mixing GelMA with PCL remains unresolved (Figure 1C, top left corner).

Nerve guidance conduits (NGCs) represent the most convenient and straightforward type of artificial nerve grafts, serving as a substrate to facilitate interactions between scaffolds and host cells/tissues. They do this by inducing specific cellular and tissue immune responses and reestablishing the local microenvironment [20]. Various nanotechnological techniques have been extensively investigated to enhance these interactions, leveraging the unique effects of micro–nano-topography [21]. Among these, electrospinning has emerged as a prominent method for constructing scaffolds with distinctive micro–nano-scale features that mimic the hierarchical architecture of the extracellular matrix (ECM) [22,23].

In this study, we introduce a novel core–sheath GelMA/PCL nanofiber fabricated via electrospinning and phase separation techniques. The phase separation and subsequent polymerization during the electrospinning process result in the formation of a distinct core–sheath structure, which markedly differs from the previously reported linear composite structure (Figure 1B,C) [14,15]. This unique architecture effectively integrates the advantageous properties of both GelMA and PCL, such as wettability, degradation characteristics, and mechanical strength, thereby ensuring long-term stability of the nerve guidance conduits and enabling a controlled release system. Following implantation, axons successfully regenerated across 10 mm nerve gaps, reinnervated muscles, and reestablished neuromuscular junctions. Collectively, our findings demonstrate that the core–sheath GelMA/PCL nanofibers possess distinctive physical, chemical, and biological properties, rendering the conduit highly promising for peripheral nerve regeneration.

## 2. Materials and Methods

### 2.1. Fabrication of Core–Sheath GelMA/PCL Nanofibers Nerve Guidance Conduits

GelMA was synthesized and characterized following previously reported methods [24]. Specifically, the reaction was initiated by adding methacrylic anhydride (MA) to a 10% (*w*/*v*) gelatin solution under stirring conditions until the final concentration of MA reached 6% (*v*/*v*). The mixture was then maintained at 50 °C with vigorous stirring for 3 h. Upon completion of the reaction, which was terminated by dilution with double-distilled water, the resulting solution was dialyzed against double-distilled water using 12–14 kDa cut-off dialysis tubing for one week to ensure complete removal of unreacted MA and other byproducts. Finally, the dialyzed solution was lyophilized and stored until further use.

**Figure 2:**
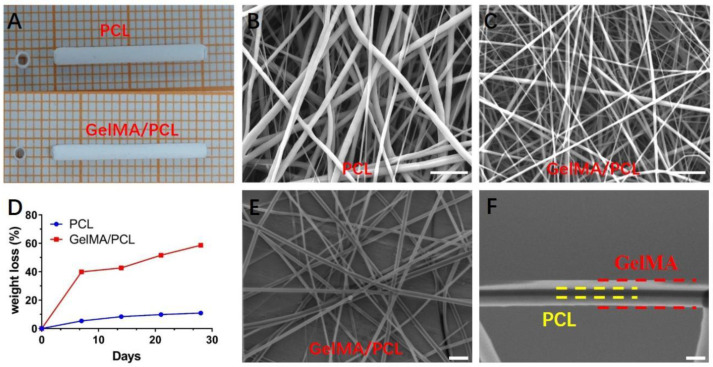
(**A**) Schematic illustration of the electrospinning process and the core–sheath structure of GelMA/PCL nanofibers; (**B**,**C**) SEM images of pure PCL nanofibers and core–sheath GelMA/PCL nanofibers. Scale bar = 10 μm. (**D**) In vitro degradation behavior of pure PCL nanofibers and core–sheath structured GelMA/PCL nanofibers over a 4-week period. (**E**) STEM image of core–sheath structured GelMA/PCL nanofibers. The red and yellow regions correspond to GelMA, while the yellow region represents PCL. Scale bar = 1000 nm. (**F**) is an enlarged image of (**E**). Scale bar = 100 nm.

A solution was prepared by dissolving 5% *w*/*v* GelMA, 5% *w*/*v* PCL, and 0.5% *w*/*v* Irgacure 2959 (photoinitiator) in trifluoroethanol (TFE). The pH was adjusted, and the mixture was vigorously stirred overnight until a clear and transparent solution was obtained. This solution was then loaded into a 10 mL syringe equipped with a 21-gauge stainless-steel needle. Electrospinning was performed using an IonBeam WL-2C electrospinner (Beijing, China) under the following conditions: UV light intensity of 9 W, flow rate of 2 mL/h, needle-to-collector distance of 15 cm, applied voltage of 15 kV, temperature range of 30–40 °C, and relative humidity of 30–40%. The nanofibers were collected on a rotating mandrel consisting of a 1.2 mm diameter stainless-steel rod rotating at 800 rpm. After electrospinning, the nanofibers and their nerve guidance conduits (NGCs) were dried under vacuum for at least 3 days before removing the stainless-steel rods. Pure PCL nanofibers and their NGCs, used as controls, were prepared using the same method (Figure 2A).

### 2.2. Characterization of Core–Sheath GelMA/PCL Nanofibers

Scanning electron microscopy (SEM, JEOL JSM-7900F, Akishima, Japan) was employed to examine the surface morphology and determine the average diameter and standard deviation of the fibers. Quantitative analysis of individual fiber dimensions was conducted using Image J software Version 1.8.0 (n = 50).

The core–sheath structure was characterized using 1% phosphotungstic acid and fluorescence isothiocyanate (FITC) staining. Specifically, the core–sheath GelMA/PCL nanofibers were immersed in 1% phosphotungstic acid solution and analyzed via scanning transmission electron microscopy (STEM, JEOL JSM-7900F, Okinawa, Japan). The isothiocyanate group of FITC can specifically react with the primary amino groups on the lysine residues in GelMA molecules, forming stable thiourea bonds, thereby achieving a strong covalent binding. FITC exhibits poor adhesion to polycaprolactone (PCL) owing to PCL’s inherent chemical inertness—its aliphatic polyester backbone lacks reactive functional groups (e.g., amines, carboxyls, or hydroxyls) capable of covalently coupling with FITC. Consequently, FITC binding is restricted to weak, non-covalent interactions (e.g., van der Waals forces and hydrophobic associations), which are readily disrupted during routine washing or cell culture procedures, leading to substantial signal loss. For FITC staining, both core–sheath GelMA/PCL nanofibers and pure PCL nanofibers were incubated with FITC solutions for 1 h, followed by two washes with deionized water. The FITC-labeled nanofibers were subsequently examined using laser scanning confocal microscopy (Leica TCS-SP8 STED 3X, Wetzlar, Germany).

The surface wettability of core–sheath GelMA/PCL nanofibers and pure PCL nanofibers was evaluated by measuring the water contact angle (WCA). The WCA was determined using a contact angle measurement platform (Dataphysics DCAT21, Filderstadt, Germany) with 5 μL droplets of distilled water.

The mechanical properties of core–sheath GelMA/PCL nanofibers and pure PCL nanofibers were evaluated using a biomaterials testing instrument (CARE, IBTC-5000, Tianjin, China) at a constant tensile rate of 0.1 mm/min under ambient conditions. All scaffold samples were fabricated in a dumbbell shape with rectangular dimensions of 13 × 5 mm^2^ (length × width) and an average thickness of approximately 200 um, as measured by a digital screw micrometer. At least five samples were tested for each group. The mechanical properties, including elastic modulus, ultimate tensile strength, and fracture strain, were subsequently calculated.

The in vitro degradation test was conducted to evaluate the performance of the controlled release system. Predetermined weights of nanofibers were immersed in 1 mL of 0.5 U/mL collagenase type II solution (Life Technologies, catalog number: 17101-015, Waltham, MA, USA) and incubated at 37 °C for 1, 2, 3, and 4 weeks. The solution was refreshed every 3 days. At each time point, the samples were retrieved, rinsed with ultrapure water, lyophilized, and reweighed. The weight loss was calculated using the following formula:

Weight loss (%) = (M_0_ − Mt)/M_0_ × 100%

M_0_ represents the initial weight of the sample, while Mt represents the residual weight of the material after degradation over different periods of time. Data for each time point were derived from six replicate samples per group.

### 2.3. Cell Viability, Biocompatibility Assessment, and Expression Analysis of Dedifferentiation-Associated Genes

The RSC96 Schwann cell line was seeded onto core–sheath GelMA/PCL nanofibers and pure PCL nanofibers at a density of 3 × 10^4^ cells/cm^2^ and cultured for 3 days. The morphology of the cell-laden scaffolds was fixed using 2.5% (*v*/*v*) glutaraldehyde, dried in a critical point dryer (Leica CPD 300, Wetzlar, Germany), and subsequently analyzed by scanning electron microscopy (SEM).

Cell proliferation was assessed using the CCK-8 assay (Cell Counting Kit-8, Beyotime, C0039, Shanghai, China), with three replicates performed for each sample. Scaffold samples, including both core–sheath GelMA/PCL nanofibers and pure PCL nanofibers, were washed three times with PBS buffer and then incubated in 1 mL of complete growth medium (CGM; Dulbecco’s Modified Eagle Medium supplemented with 10% heat-inactivated fetal bovine serum, 100 units/mL penicillin, and 100 μg/mL streptomycin) at 37 °C under 5% CO_2_ and 95% relative humidity for 24 h. The extraction media were collected and stored for subsequent use. For the proliferation assay, RSC96 cells were seeded into a 96-well plate at a density of 5000 cells/well and allowed to adhere for 1 h. Subsequently, 100 μL of fresh culture medium containing the respective extraction media from the core–sheath GelMA/PCL nanofibers or pure PCL nanofibers was added to each well (Control group, GelMA/PCL group, and PCL group). At each time point, 10 μL of CCK-8 solution was added to each well, followed by an additional 1-h incubation. Absorbance at 450 nm was measured using a microplate reader (Bio-Rad Laboratories, iMark, Shanghai, China).

To investigate the expression of Schwann cell differentiation-associated genes—Krox24, Cyclin D1, p75, and Sox2—in RSC96 cells cultured on hybrid nanofibrous scaffolds, quantitative reverse transcription polymerase chain reaction (qRT–PCR) was performed. RSC96 rat Schwann cells were seeded onto the scaffolds at a density of 3 × 10^4^ cells/cm^2^ and maintained under standard culture conditions for 72 h. Experimental groups included: (i) Control—RSC96 cells cultured on tissue-culture-treated polystyrene plates; (ii) PCL—cells seeded on electrospun pure PCL nanofibrous scaffolds; and (iii) GelMA/PCL—cells seeded on Core–sheath GelMA/PCL nanofibrous scaffolds. Total RNA was extracted using the TIANGEN RNAprep Pure Cell/Bacteria Kit (TIANGEN Biotech, Beijing, China) and treated with DNase I to eliminate genomic DNA contamination. First-strand cDNA synthesis was carried out using the RevertAid First Strand cDNA Synthesis Kit (Thermo Scientific, Mississauga, ON, Canada) with oligo(dT) primers. qRT–PCR amplification was performed in triplicate on an ABI StepOnePlus Real-Time PCR System (Applied Biosystems, Waltham, MA, USA) using PowerUp SYBR Green Master Mix (Thermo Fisher Scientific, Waltham, MA, USA). Amplification specificity was confirmed by analysis of melting curves and agarose gel electrophoresis of representative amplicons. Data were analyzed using Bio-Rad CFX Maestro Software (v1.1). GAPDH served as the endogenous reference gene, and relative mRNA expression levels were calculated using the 2^(−ΔΔCt)^ method. Primer sequences for all target genes and GAPDH are listed in Table S1.

### 2.4. Surgical Procedure

All experimental designs and protocols involving animals were approved by the Animal Ethics Committee of Peking University People’s Hospital, Beijing, People’s Republic of China and complied with the recommendations of the academy’s animal research guidelines (see the Form S1 in the Supplementary Materials). Animals were randomly assigned to four groups (n = 6 per group): (1) core–sheath GelMA/PCL nanofiber nerve guidance conduits (NGCs) group (GelMA/PCL group); (2) PCL nanofiber NGCs group (PCL group); (3) autograft group; and 4) non-operated control group (Normal group). The surgical procedure followed previously described methods [25]. Adult female Sprague-Dawley rats (200–250 g) were anesthetized with 5% isoflurane for induction and maintained at 1.5–2.0% isoflurane during surgery. The surgical site was prepared by shaving, and the skin was incised. Blunt dissection was used to separate the thigh muscles, exposing the right sciatic nerve. A 10 mm gap was created by transecting the nerve at the mid-thigh, and the NGCs or autografts were sutured into place using a 10-0 Nylon monofilament. For the GelMA/PCL and PCL groups, 13–15 mm NGCs were used to bridge the 10 mm gap, accounting for the 1.5–2 mm overlap required at each end. Autologous nerve grafts were reimplanted via epineural coaptation. Muscles and skin were closed with 4-0 nylon sutures. Postoperative observations were conducted at week 12.

### 2.5. Walking Track Analysis

The functional nerve recovery of the animals was assessed using the Catwalk XT system (Noldus, Wageningen, The Netherlands) at 12 weeks post-surgery. Specifically, animals were permitted to walk along the walkway for data acquisition. Subsequently, key parameters of the footprints, including stance phase, maximum contact area, duty cycle, and relative paw position, were analyzed. Both the non-operated left hind limb and the operated right hind limb were evaluated.

### 2.6. Electrophysiological Assessment

To evaluate the functional recovery of the repaired and regenerated nerve, electrophysiological tests were conducted to measure the compound muscle action potential (CMAP) and motor nerve conduction velocity (MNCV) using a VIASYS Synergy 5CH device (Oxford Instruments, Abingdon, UK). Twelve weeks post-surgery, under anesthesia, both the right sciatic nerve (operated) and the left sciatic nerve (non-operated) were exposed. A monopolar stimulating electrode was placed at both the proximal and distal ends of the nerve segment, with the distance between these points carefully measured. Recording electrodes were positioned in the tibialis anterior muscle.

### 2.7. Morphometric Evaluation of Axonal Regeneration

The nerves, containing NGCs, were immediately dissected following transcardial perfusion. All samples were fixed in 4% paraformaldehyde (PFA) and subsequently soaked in 30% sucrose in PBS until they sank.

For immunofluorescence analysis, longitudinal and coronal sections of the nerve samples, each 8 μm thick, were prepared using cryosectioning techniques. Middle sections of the regenerated nerves were specifically processed for analysis. Sections from each specimen were collected and incubated with the following primary antibodies: NF200 (Sigma, N0142, St. Louis, MO, USA, 1:400) to label axons, and S-100 (Abcam, ab52642, Waltham, MA, USA, 1:500) to label terminal Schwann cells. Secondary antibodies used were goat anti-rabbit IgG H&L (ZSGB-Bio, ZF-0516, Beijing, China, 1:500) and goat anti-mouse IgG H&L (ZSGB-Bio, ZF-0512, Beijing, China, 1:500). Quantitative and qualitative assessments of the nerve samples were conducted using laser scanning confocal microscopy (Leica, TCS-SP8 STED 3X, Wetzlar, Germany). Three coronal sections were selected for calculating the nerve fiber count.

Axonal regeneration in the mid-regions of the regenerated sciatic nerves was investigated using transmission electron microscopy (Thermo Scientific (formerly FEI), Hillsboro, OR, USA). Samples were initially fixed with 2.5% glutaraldehyde in 0.1 M phosphate buffer (pH 7.4) for 1 h, followed by post-fixation in 2% OsO4 for 1 h at room temperature. After several rinses with distilled water, the specimens were dehydrated through a graded alcohol series and subsequently embedded in Epon 812 resin (SPI Supplies, West Chester, PA, USA). Ultra-thin sections (70 nm) were prepared using an ultramicrotome and observed under the transmission electron microscope (FEI, Tecnai G2 Spirit) at 120 kV. For each specimen, at least five different regions were analyzed. The G-ratio, which quantitatively assesses myelin sheath thickness, was measured using Image J software. From each animal (n = 6), three non-consecutive ultrathin sections (inter-section distance ≥ 100 μm) were randomly selected. For each section, five non-overlapping electron micrographs were acquired at ×20,000 magnification under transmission electron microscopy (TEM). Only intact, transversely oriented axons with fully preserved myelin sheaths—excluding those exhibiting tilt, folding, or myelin disruption—were included for morphometric analysis using ImageJ software. A minimum of 75 axons per animal was quantified.

### 2.8. Assessment of Angiogenesis

Longitudinal 8 μm thick cryosections of the nerve samples were prepared and subsequently utilized for vWF immunofluorescence assays. Primary antibodies included vWF (Agilent, A008229-2, Santa Clara, CA, USA, diluted 1:5000) and S-100 (Abcam, ab4066, Waltham, MA, USA, diluted 1:500). Secondary antibodies comprised goat anti-rabbit IgG H&L (ZSGB-Bio, ZF-0516, Beijing, China, diluted 1:500) and goat anti-mouse IgG H&L (ZSGB-Bio, ZF-0512, Beijing, China, diluted 1:500).

### 2.9. Target Muscles Evaluation

After 12 weeks of implantation, the tibialis anterior and gastrocnemius muscles were carefully dissected and weighed. Both the experimental side and the contralateral side (non-operated) were measured. The relative weights were expressed as percentages, in accordance with previously published methods [26].

$$\text{Muscles}\;\text{weight}\%=\frac{\text{muscles}\;\text{weight}\;\text{of}\;\text{the}\;\text{operated}\;\text{leg}}{\text{muscles}\;\text{weight}\;\text{of}\;\text{the}\;\text{unoperated}\;\text{leg}}\times 100\%$$

After initial weighing, the muscles were fixed overnight in 4% paraformaldehyde (PFA) and subsequently soaked in 30% sucrose in PBS until they sank.

### 2.10. Assessment of the Neuromuscular Junction of Reinnervated Muscles

To analyze the neuromuscular junctions of reinnervated muscles, immunofluorescence staining was performed on 40 µm thick longitudinal sections of tibialis anterior muscles from six animals in each category. The sections were stained with the following markers: NF200 (Sigma, N0142, 1:400) for axons, S-100 (Abcam, ab52642, 1:500) for terminal Schwann cells, alpha-Bungarotoxin–tetramethylrhodamine (α-Bung, Sigma, B12423, St. Louis, MO, USA, 1:500) to visualize acetylcholine receptors at the neuromuscular junctions, and SV2 antibody (synaptic vesicle protein 2, Abcam, ab32942, Waltham, MA, USA, 1:1000) for synaptic vesicles at neuromuscular junctions. Laser scanning confocal microscopy was utilized for imaging. Quantification of reconstructed neuromuscular junctions followed previously reported methods [27]. For this analysis, at least six random sample areas per animal were selected for quantification. The parameters used to quantify the rebuilding of neuromuscular junctions in the affected tibialis anterior muscles (n = 6 for each category) included: (1) the percentage of S100+ terminal Schwann cells associated with acetylcholine receptors (AChR), calculated by dividing the number of co-localized S100+ terminal Schwann cells and α-Bung+ NMJs by the total number of α-Bung+ NMJs; (2) the percentage of NF200+ axons innervating acetylcholine receptors (AChR), determined by dividing the number of co-localized NF200+ axons and α-Bung+ NMJs by the total number of α-Bung+ NMJs; and (3) the percentage of co-localization between SV2+ synaptic vesicles and acetylcholine receptors (AChR), assessed by dividing the number of co-localized SV2+ synaptic vesicles and α-Bung+ NMJs by the total number of α-Bung+ NMJs.

### 2.11. Statistical Analysis and Data Availability Statement

This study employed one-way analysis of variance (ANOVA) to test for statistically significant differences among group means. A significant ANOVA result was followed by pairwise post hoc comparisons using Tukey’s honestly significant difference (HSD) test, which controls the family-wise error rate under balanced or near-balanced designs. Prior to ANOVA, the assumptions of normality and homogeneity of variances were rigorously verified: normality was assessed using the Shapiro–Wilk test and corroborated visually with Q–Q plots; homogeneity of variances was evaluated using Levene’s test. All groups satisfied both assumptions, justifying the use of parametric inference. Data are presented as mean ± standard error of the mean (SEM), and statistical significance was defined as *p* < 0.05.

Given the inherent nature of the interventions—specifically, the physical distinction between surgical and sham-surgical procedures—blinding of subjects and intervention implementers was not feasible. To minimize assessment bias, outcome assessors and data analysts were blinded to group allocation. Animal identifiers and treatment assignments were managed exclusively by a researcher uninvolved in data collection or analysis. All behavioral test videos, histological tissue section images, and radiographic imaging datasets underwent rigorous anonymization (i.e., removal of all animal- and group-specific metadata) prior to evaluation by blinded assessors.

## 3. Results

### 3.1. Fabrication and Characterization of a Core–Sheath GelMA/PCL Nanofiber Nerve Guidance Conduit

Generally, both GelMA and PCL added to trifluoroethanol exhibit significant phase separation (Figure 1C, top left corner), which aligns with the notion that kinetic factors hinder the formation of a fully homogeneous material in this two-phase (hydrophilic and hydrophobic) system [27,28]. In this study, a mixture of 5% *w*/*v* PCL and 5% *w*/*v* GelMA (1:1) was stabilized into a transparent and homogeneous solution through pH adjustment (Figure 1C, left bottom). This is a critical step for successful electrospinning, indicating the elimination of phase separation. We hypothesize that pH modification alters the charge distribution of GelMA, allowing its hydrophobic groups to form weak bonds with PCL.

Figure 1C illustrates a schematic of the electrospinning setup utilized for fabricating the nanofibers. A specially designed collector, comprising a 1.2 mm stainless-steel rod and a fixture, was employed to produce a hollow conduit. The nerve guidance conduits (NGCs) were obtained by removing the stainless-steel rod. As depicted in Figure 2A–C, the core–sheath GelMA/PCL nanofiber NGCs exhibited an internal diameter of 1.2 mm and a wall thickness of approximately 200 μm. This internal diameter is appropriate for bridging a rat sciatic nerve defect, given that the width of a rat’s sciatic nerve is approximately 1.2 mm. Moreover, the thin-walled structure facilitates controlled diffusion of nutrients, gases, metabolites, wound fluid, bio-information, and other substances [23,29].

SEM images of pure PCL nanofibers and core–sheath GelMA/PCL nanofibers are presented in Figure 2B,C. The nanofibers were randomly oriented, with average diameters of 605 nm for core–sheath GelMA/PCL nanofibers and 1290 nm for pure PCL nanofibers (Figure S1B). The diameter distribution of core–sheath GelMA/PCL nanofibers was narrow, whereas that of pure PCL nanofibers was broad (Figure S1A). These findings indicate that the incorporation of GelMA significantly affects both the diameter size and distribution of the nanofibers compared with pure PCL nanofibers. The smaller diameter of the nanofibers closely mimics the scale of ECM fibers and enhances the diffusion of degradation products/byproducts due to increased surface area [30]. Therefore, we hypothesize that core–sheath GelMA/PCL nanofibers could serve as an effective ECM mimic for peripheral nerve regeneration.

We observed that phase separation during the electrospinning process resulted in the formation of a core–sheath structure. Specifically, GelMA forms a hydrophilic phase within the aqueous electrospinning solution, while PCL forms a hydrophobic phase. Initially, pH adjustments disrupt phase separation; however, as the pH returns to its original state during electrospinning, phase separation reoccurs. This re-emergence of phase separation facilitates UV-induced polymerization of GelMA, leading to its spontaneous distribution on the surface of PCL, thereby forming a core–sheath structure (Figure 1).

To directly verify the unique core–sheath structure, core–sheath GelMA/PCL nanofibers were immersed in 1% phosphotungstic acid solution, which stained the GelMA component. Phosphotungstic acid, acting as a positive stain, reacts with surface functional groups of GelMA such as hydroxyl, carboxyl, and amine groups. As shown in Figure 2E,F, the white areas representing GelMA are distributed on the dark areas representing PCL, confirming the presence of a core–sheath structure. Additionally, to visualize the GelMA polymer on the surface of PCL, we incubated the core–sheath GelMA/PCL nanofibers with fluorescence isothiocyanate (FITC)-labeled GelMA. As illustrated in Figure S2, the smooth and uniformly fluorescent green structures indicate that the PCL core is coated and crosslinked by FITC-GelMA, consistent with previous studies [31,32]. In contrast, no such fluorescent green structures were observed in pure PCL nanofibers (Figure S2A). These results suggest that phase separation during the electrospinning process induces the formation of a core–sheath structure, which is significantly different from the linear composite structure reported previously (Figure 1A) [14,15]. More importantly, this core–sheath structure substantially influences the characteristics of the nanofibers compared with the previously reported linear composite structure [14,15].

As illustrated in Figure S3A, the water contact angle of pure PCL nanofibers was measured at 84.3°, consistent with the established understanding that PCL is hydrophobic. In contrast, GelMA is known for its hydrophilic nature, typically exhibiting a water contact angle close to 0°. As shown in Figure S3B, the water contact angle of core–sheath GelMA/PCL nanofibers was also 0°, indicating that the outer layer of GelMA significantly enhances the wettability of the hybrid nanofibers (the experimental procedure is documented in the Video S1 in the Supplementary Materials). This observation not only reflects the surface properties but also indirectly confirms the core–sheath structure.

Generally, pure GelMA hydrogels exhibit weak mechanical properties, whereas PCL possesses excellent mechanical strength. Therefore, incorporating PCL into GelMA aims to enhance the mechanical performance of the hybrid material. As demonstrated in Figure S4A, compared with pure PCL nanofibers, the stress of core–sheath GelMA/PCL nanofibers increased, while the strain decreased. Specifically, the average elastic modulus of core–sheath GelMA/PCL nanofibers was 8.18 MPa, significantly higher than that of pure PCL nanofibers at 2.78 MPa (Figure S4B). The fracture strain of core–sheath GelMA/PCL nanofibers was approximately 41.8%, markedly lower than the 98.5% observed in pure PCL nanofibers (Figure S4C). Additionally, the ultimate tensile strength of core–sheath GelMA/PCL nanofibers reached around 3.69 N, substantially higher than the 2.70 N recorded for pure PCL nanofibers (Figure S4D). These results collectively indicate that the incorporation of PCL significantly improves the mechanical properties of core–sheath GelMA/PCL nanofibers, suggesting their potential suitability for neural graft applications.

To evaluate the impact of the core–sheath structure on the degradation properties of nanofibers, we exposed core–sheath GelMA/PCL nanofibers and pure PCL nanofibers to collagenase digestion solutions for 4 weeks. GelMA is susceptible to degradation by collagenase due to the presence of MMP-sensitive sites. In contrast, PCL degrades more slowly because of its hydrolytically labile aliphatic ester linkages. The core–sheath GelMA/PCL nanofibers exhibited rapid initial degradation within the first 7 days (up to 40%), primarily attributed to the degradation of GelMA. However, during the subsequent 21 days, the degradation rate significantly decreased, reflecting the combined degradation of both GelMA and PCL (Figure 2D). In contrast, pure PCL nanofibers degraded at a consistently slow rate throughout the entire period (as reported to take 2–3 years). Previous studies have indicated that degradation products and byproducts of GelMA can influence nerve-associated cell/stem cell behaviors [33,34]. Additionally, the prolonged degradation of PCL provides long-term structural support for regenerated nerves, fostering a microenvironment conducive to cell interaction. Therefore, these findings indicate that the core–sheath architecture strategically exploits the distinct degradation kinetics of gelatin methacryloyl (GelMA) and poly(ε-caprolactone) (PCL) to establish a spatiotemporally controlled gradient release system while concurrently delivering mechanical support—both of which are conducive to peripheral nerve regeneration. It should be emphasized that the current release profile was characterized under simulated (e.g., in vitro enzymatic or pH-shifted) or accelerated degradation conditions; thus, the observed controlled-release behavior reflects an engineered design principle rather than in vivo performance. Comprehensive validation of the system’s pharmacokinetic fidelity, temporal release precision, and therapeutic efficacy under physiological conditions remains a key objective for future studies.

### 3.2. The Behavior of Schwann Cells After Transplantation In Vivo

**Figure 3:**
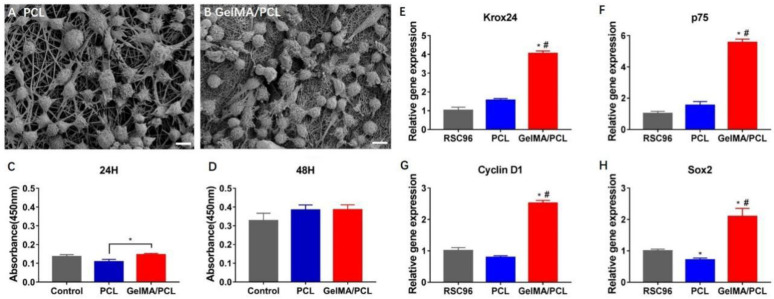
SEM images of RSC96 cells cultured on pure pcl nanofibers (**A**) and core–sheath GelMA/PCL nanofibers (**B**) after 3 days, respectively, (**C**,**D**) Cell Proliferation after 24 h and 48 h of Culture (**E**–**H**) The gene expression of Krox24, p75, Cylin D1 and Sox2 genes. * *p* represents the comparison between the RSC96 group and the PCL or GelMA/PCL group; # *p* represents the com-parison between the PCL group and the GelMA/PCL group. * p < 0.05, *# p* < 0.05. Error bar = s.e.m. n = 3.

Notably, the interaction between cells and scaffolds plays a critical role in determining cell adhesion and proliferation, which subsequently influences cell fate. Rat Schwann cells (RSC96) were cultured on core–sheath GelMA/PCL nanofibers and pure PCL nanofibers for 3 days to evaluate their adhesion and spreading on these substrates. As shown in Figure 3A,B, RSC96 cells fully covered the surfaces of both core–sheath GelMA/PCL nanofibers and pure PCL nanofibers, demonstrating excellent adhesion with the nanofibers and surrounding cells. Cell proliferation was assessed using the CCK8 assay (Figure 3C,D). The GelMA/PCL group exhibited a similar trend to the Control group (used as a positive control), but differed significantly from the PCL group. Interestingly, this difference diminished after 48 h, likely due to the time required for cells to adapt to the lower wettability of PCL. Therefore, the incorporation of GelMA on the surface of PCL does not significantly affect cell adhesion and proliferation compared with PCL alone. Interestingly, the majority of dedifferentiation-related genes showed the highest expression levels in the GelMA/PCL group, whereas the lowest expression levels were observed in the PCL and RSC96 groups (Figure 3E–H).

### 3.3. Functional and Morphological Evaluation of Regenerated Nerves

**Figure 4:**
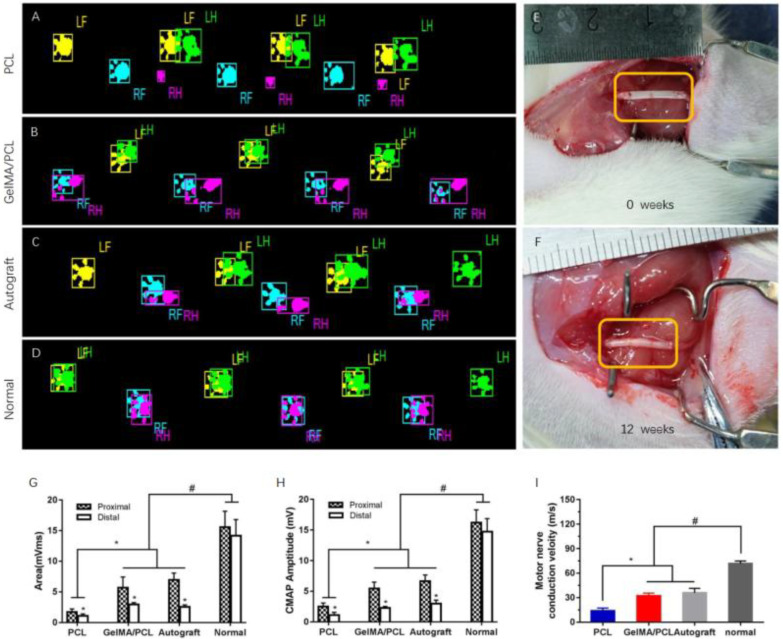
(**A**–**D**) Footprints and footfall patterns of the PCL group, GelMA/PCL group, Autograft group, and Normal group after 12 weeks of implantation, respectively. (**E**,**F**) Intraoperative images of core–sheath GelMA/PCL nanofibers nerve guidance conduit bridging a 10-mm sciatic nerve defect in rats at 0 weeks (**E**) and 12 weeks (**F**). The orange box marks the implantation area. (**G**–**I**) Electrophysiology and behavioral outcomes. CMAP Areas (**H**) and Amplitudes (**G**), * *p* < 0.05 between proximal and distal of the sciatic nerve. * *p* < 0.05, # *p* < 0.05. Error bar = s.e.m. (**I**) Motor nerve conduction velocities. * *p* < 0.05, # *p* < 0.05. Error bar = s.e.m. n = 6.

Next, we utilized core–sheath GelMA/PCL nanofibers as a nerve guidance conduit (NGC) to repair a 10 mm sciatic nerve defect in Sprague-Dawley (SD) rats. All surgical incisions healed uneventfully without complications or infections. Figure 2A illustrates the morphologies of the core–sheath GelMA/PCL nanofiber nerve guidance conduits immediately post-implantation and 12 weeks post-surgery. The conduits effectively bridged the nerve gaps via neurorrhaphy, creating a relatively enclosed environment (Figure 4E). This microenvironment facilitates the permeation of nutrients, oxygen, and growth factors at appropriate concentrations while inhibiting neuroma formation, scar tissue development, myofibroblast infiltration, and collateral axon sprouting. After 12 weeks of implantation, the nerves proximal and distal to the core–sheath GelMA/PCL nanofiber conduits remained robust and intact, with no signs of atrophy, neuroma, or scar formation, demonstrating favorable biocompatibility (Figure 4F). Additionally, several blood vessels and fibrous connective tissues were observed on the surface of the conduits, indicating their excellent biocompatibility and potential pro-angiogenic properties. In summary, these results suggest that core–sheath GelMA/PCL nanofiber nerve guidance conduits (NGCs) provide sustained support for nerve regeneration and exhibit superior biocompatibility.

To evaluate the functional recovery of all experimental rats, walking track analysis was conducted. Proper walking necessitates coordinated function involving sensory input, motor response, and cortical integration. Walking track analysis offers a non-invasive method to assess the functional status of the sciatic nerve during the regeneration process. During the test, the walking tracks of the GelMA/PCL group and Autograft group were comparable to those of the Normal group (non-operated rats), whereas some rats in the PCL group exhibited difficulty placing their feet on the ground due to contracture formation on the right hind limb (the operated side) (see Video S2 in the Supplementary Materials ). The footprints and footfall patterns of the PCL group, GelMA/PCL group, and Autograft group at 12 weeks post-implantation (Left Front, LF; Left Hind, LH; Right Front, RF; Right Hind, RH) showed differences compared with those of the Normal group (Figure 4A–D). Consequently, several parameters were analyzed, including stance, maximum contact area, and duty cycle, which depend on the pressure exerted by the paw during locomotion and can be used to assess mechanical allodynia. As illustrated in Figure S5, both the GelMA/PCL group and the Autograft group exhibited similar levels of mechanical allodynia, whereas the PCL group demonstrated significantly higher pain sensitivity. Specifically, rats tended to place their hind paws in the previous positions of their forepaws, indicating that Relative Paw Position is a sensitive parameter for outcome assessment. As shown in Figure S5D, the relative positions of the right hind paws in the GelMA/PCL group were smaller compared with those in the PCL group, and not significantly different from those in the Autograft group, but significantly different from those in the Normal group. Additionally, the relative positions of the left fore and hind paws in the PCL group were also affected due to inadequate regeneration of the right-side sciatic nerve (Figure S5E). In summary, these findings suggest that core–sheath GelMA/PCL nanofiber NGCs can enhance the recovery of sensory and motor functions, achieving results comparable to the gold standard autograft.

Since electrophysiological evaluation is the most sensitive indicator of functional recovery in sciatic nerve regeneration, compound muscle action potential (CMAP) and motor nerve conduction velocity (MNCV) were recorded at 12 weeks postoperatively (Figure 4G–I and Figure S6). CMAP indirectly reflects the number of regenerated motor nerve fibers and the rate of muscle reinnervation. The peak amplitude and area of CMAPs in the GelMA/PCL group were significantly higher than those in the PCL group but not substantially different from those in the Autograft group, while being significantly lower than those in the Normal group (the contralateral uninjured side). These results suggest that core–sheath GelMA/PCL nerve guidance conduits (NCGs) can enhance the number of regenerated motor nerve fibers and the rate of muscle reinnervation. Additionally, in the PCL, GelMA/PCL, and Autograft groups, proximal CMAPs (peak amplitude/area) were significantly higher than distal CMAPs, whereas no difference was observed between proximal and distal CMAPs in the Normal group. This indicates that the number of proximally regenerated motor nerve fibers exceeds that of distally regenerated fibers. The MNCV is influenced by fiber diameter, axon diameter, and myelin thickness. The mean MNCV in the Normal group was 71.7 m/s, compared with 14.0 m/s in the PCL group, 32.2 m/s in the GelMA/PCL group, and 35.9 m/s in the Autograft group (Figure 4I), suggesting that the GelMA/PCL group has well-developed fiber diameter, axon diameter, and myelin thickness.

**Figure 5:**
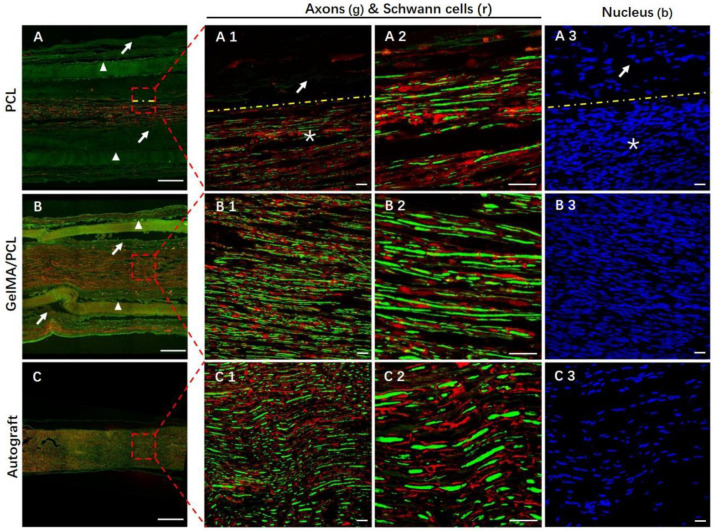
Immunofluorescent analysis of nerve regeneration in the transverse cross-section at the mid-implantation site. (**A**) PCL group; (**B**) GelMA/PCL group; (**C**) Autograft group. Triple immunostaining of the nucleus (blue), axons (green), and Schwann cells (red). White triangles indicate pure PCL nanofibers nerve guidance conduits or core–sheath GelMA/PCL nanofibers nerve guidance conduits. White arrows indicate the axon/Schwann cell-depleted areas. (**A**–**C**): Scale bar = 250 μm. (**A1**–**C1**,**A2**–**C2**,**A3**–**C3**): Scale bar = 25 μm. * denotes the same anatomical location as that indicated by the asterisk in panel A1—specifically, the region of regenerating nerve tissue.

To directly verify the axon regrowth and remyelination facilitated by the implants, we obtained and analyzed the implant samples using immunofluorescence staining (Figure 5 and Figure S7). The axon/Schwann cell-depleted area is either empty or occupied by non-neuronal cells (such as fibroblasts, blood vessels, macrophages, etc.) and the degraded products of conduit materials. Therefore, a smaller axon/Schwann cell-depleted area indicates better nerve regeneration. As shown in Figure 5 and Figure S7, axon/Schwann cell-depleted areas were observed both around and inside the conduits in the GelMA/PCL group and PCL group, whereas in the Autograft group, these areas were primarily located around the autograft (white triangles indicate the conduits). Notably, the axon/Schwann cell-depleted area in the GelMA/PCL group was significantly smaller than that in the PCL group, suggesting superior nerve regeneration outcomes in the GelMA/PCL group. In all groups, regenerated NF200+ axons were almost entirely co-localized with S100+ Schwann cells, indicating well-preserved nerve structures. As illustrated in Figure 5(A1–C1) and Figure S7(A3–C3), compared with the Autograft group, a higher density of nuclei was observed in the regenerated nerve area, suggesting an extensive multi-cellular response involving both neuronal and non-neuronal cells. The pattern of nerve regeneration explains this phenomenon: the regenerated nerves in the conduit groups must fill the hollow conduits independently, whereas the regenerated nerves in the Autograft group can utilize their pre-existing structures and environments (Indeed, certain neural structures have sustained damage; however, their basal lamina tubes remain intact). As illustrated in Figure S7G, the number of axons in the GelMA/PCL group was greater than that in the PCL group but lower than that in the Autograft group. The distinct patterns of nerve regeneration influence the area occupied by regenerated axons, which is the primary reason for these differences. However, we hypothesize that the axon numbers in the GelMA/PCL group will eventually match those in the Autograft group.

**Figure 6:**
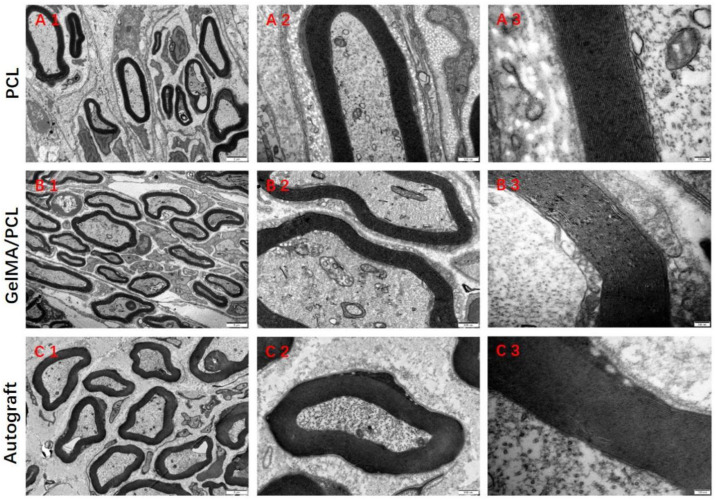
TEM images of transverse sections obtained from the mid-region of the harvested nerve grafts. (**A1**–**C1**): Scale bar = 2 μm, (**A2**–**C2**): Scale bar =500 μm, (**A3**–**C3**): Scale bar =100 μm.

The TEM images provided more detailed insights into the maturity of nerve conduction velocity and the degree of myelination in regenerated nerves, as indicated by axonal area and myelin sheath thickness. The axonal density in the GelMA/PCL group was higher than that in the Autograft and PCL groups (Figure 6). The axon diameter in the GelMA/PCL group was comparable to that of the Autograft group, whereas the axon diameters in both the GelMA/PCL and PCL groups were lower than those of the Autograft group (Figure 6). The trend in myelin sheath thickness in the GelMA/PCL group was similar to that observed in the PCL group, although both the GelMA/PCL and PCL groups exhibited reduced myelin sheath thickness compared with the Autograft group (Figure 6). The G-ratio of the myelin sheath represents the ratio of inner diameter to outer diameter.

As shown in Figure S8, the G-ratio in the PCL group was the lowest among the three groups, and the GelMA/PCL group exhibited a significantly lower G-ratio compared with the Autograft group. These findings suggest that the core–sheath GelMA/PCL nanofiber NGCs can effectively enhance myelination of the regenerated nerve fibers.

**Figure 7:**
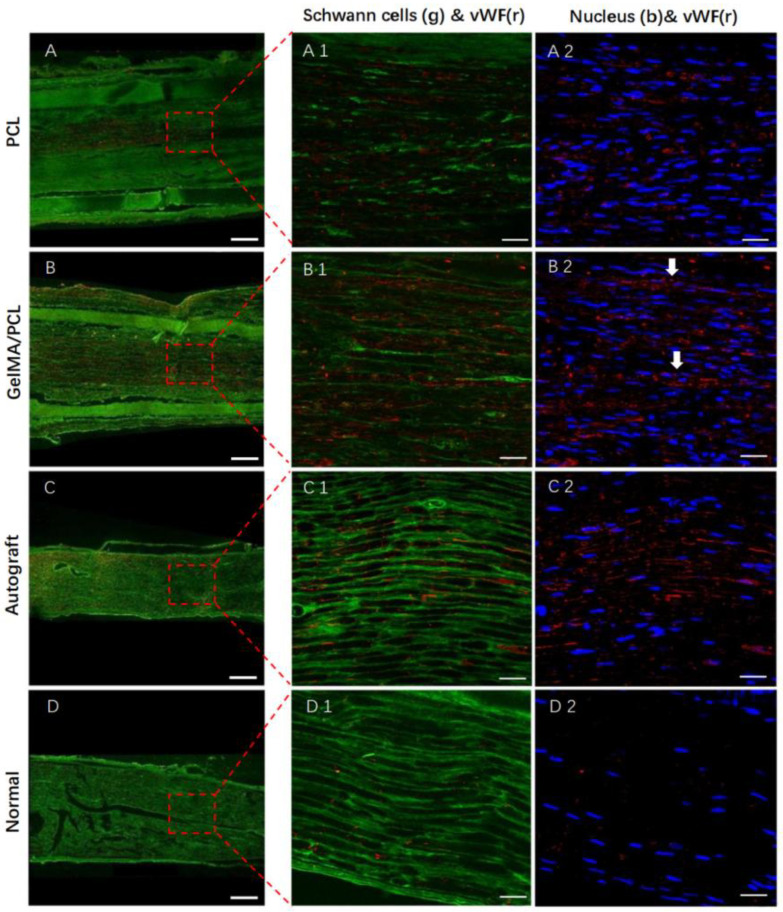
Immunofluorescent analysis of blood vessels (longitudinal section, middle of the implantation). (**A**) PCL group; (**B**) GelMA/PCL group; (**C**) Autograft group; (**D**) Normal group. Scale bar = 250 μm. Schwann cells (green), vWF (red) and nucleus (blue). White arrows indicate microvascular endothelial cells. (**A1**–**D1**,**A2**–**D2**) magnified images of (**A**–**D**), respectively. Scale bar = 25 μm.

As illustrated in Figure 7, compared with the Normal group, a significantly higher number of microvessels were observed in both the GelMA/PCL group and the Autograft group. This observation suggests that the core–sheath GelMA/PCL nanofibers (NCGs) effectively stimulate local cellular responses and promote angiogenesis. Notably, the presence of microvascular endothelial cells in conjunction with Schwann cells indicates that these structures could serve as guidance cues for Schwann cell migration.

### 3.4. Functional and Morphological Assessment of Target Muscles

**Figure 8:**
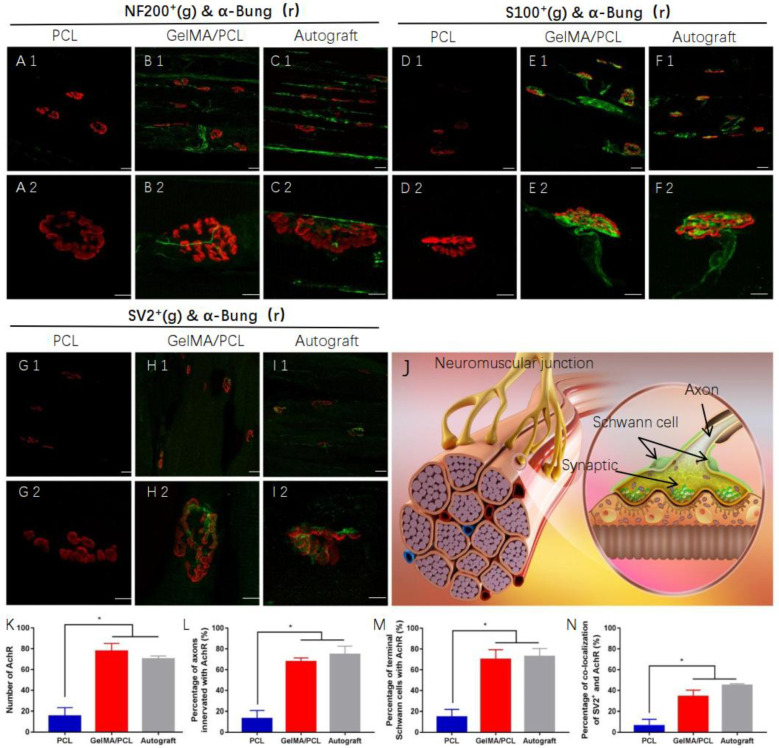
Reconstruction of functional neuromuscular junctions. (**J**) Schematic of a neuromuscular junction (NMJ). (**A1**–**C1**,**A2**–**C2**): Double immunostaining showing reinnervated axons (NF200+, green) and nicotinic acetylcholine receptors (AChR, red) at NMJs. (**D1**–**F1**,**D2**–**F2**): Double immunostaining showing terminal Schwann cells (S100+, green) and nicotinic acetylcholine receptors (AChR, red) at NMJs. (**G1**–**I1**,**G2**–**I2**): Double immunostaining showing synaptic vesicles (SV2+, green) and nicotinic acetylcholine receptors (AChR, red) at NMJs. (**A1**–**I1**), scale bar = 25 μm. (**A2**–**I2**): Magnified views of (**A1**–**I1**), respectively; scale bar = 10 μm. (**K**): Quantification of AChR-positive (α-Bung+ NMJs). * *p* < 0.05. Error bars represent s.e.m. (**L**–**N**): Percentage of axons or terminal Schwann cells co-localized with AChR, and co-localization of SV2+ and AChR. * *p* < 0.05. Error bars represent s.e.m. n = 6.

After implantation, the regenerated nerve successfully bridges the 10 mm gaps, reinnervates the muscles, and reconstructs neuromuscular junctions (NMJs). NMJs are specialized cholinergic synapses that play a crucial role in maintaining muscle function. Long-term denervation following traumatic nerve injury, which is characterized by the absence of synaptic and intercellular signaling processes, leads to a loss of NMJ plasticity and ultimately results in degeneration and atrophy. Compared with the PCL group, both the GelMA/PCL group and the Autograft group exhibited a significantly higher number of α-Bung+ (alpha-Bungarotoxin–tetramethylrhodamine) NMJs (Figure 8K). These findings indicate that while the NMJs in the PCL group have undergone degeneration and atrophy, those in the GelMA/PCL and Autograft groups have preserved their structural integrity. Furthermore, the percentage of NF200+ regenerated axons terminating at the end plates was approximately 13.1% in the PCL group, 67.6% in the GelMA/PCL group, and 74.7% in the Autograft group (Figure 8(A1–C1,A2–C2),L), which contributes to NMJ plasticity. S100+ terminal Schwann cells, myelinating cells of the peripheral nervous system known to play a key role in NMJ formation and terminal sprouting, were observed at approximately 14.7% in the PCL group, 70.0% in the GelMA/PCL group, and 72.8% in the Autograft group (Figure 8(D1–F1,D2–F2),M). These data suggest that the regenerated axons and Schwann cells in both the GelMA/PCL and Autograft groups have effectively reinnervated the end plates. More importantly, SV2+ neuromuscular junctions, which indicate functional nerve signal transmission, were present at approximately 6.25% in the PCL group, 34.1% in the GelMA/PCL group, and 44.8% in the Autograft group in the affected muscle (Figure 8(G1–I1,G2–I2),N). Therefore, these results demonstrate that the regenerated axons not only continuously regrow but also form synaptic vesicle-rich NMJs in cooperation with terminal Schwann cells.

Generally, coordinated communication between nerves and their target muscle fibers is crucial for the proper development and maintenance of neuromuscular junctions (NMJs). Disruption of this interaction can result in muscle atrophy. As shown in Figure S9, both the tibialis anterior muscle ratio and gastrocnemius muscle ratio in the GelMA/PCL group were significantly higher than those in the PCL group and were comparable to those in the Autograft group. These results suggest that, although neurodegenerative atrophy is observed across all experimental groups, both the core–sheath GelMA/PCL nanofiber nerve guidance conduits and autografts effectively alleviate this degeneration and support nerve regeneration.

## 4. Discussion

Unlike the central nervous system, peripheral nerves possess the remarkable ability to regenerate following injury [35,36]. When a peripheral nerve is severed, its continuity must be re-established to allow the growth cone of the proximal axon to extend directionally into the distal Bungner band, subsequently continuing along this band to reach the target organ and restore its original function. The microenvironment at the site of nerve injury is a critical factor influencing nerve regeneration, and reconstructing this microenvironment facilitates timely and efficient regeneration of damaged nerves [5,37].

Nerve cannula has consistently been a focal point in the research of peripheral nerve repair [21]. The nerve cannula technique restores nerve continuity by employing an artificial conduit to encase both ends of a nerve fracture. This method prevents the infiltration of fibrous scar tissue and the formation of neuromas, while promoting the precise guidance of regenerating axons through the use of distal neurochemokines, thereby facilitating nerve regeneration. In summary, the nerve cannula provides an optimal microenvironment for nerve regeneration, which is beneficial for the structural and functional recovery of nerves.

The selection of nerve cannula material is one of the core challenges in nerve cannula fabrication. A variety of materials have been utilized for constructing peripheral nerve cannulae, ranging from early options such as silicone, polytetrafluoroethylene, polycaprolactone, and chitosan, to more advanced choices like autologous veins, intestinal epithelial tissues, nerve myelin sheaths, tendons, muscles, and veins [7,20,38]. Currently, there is extensive research on synthetic degradable cannulae [20]. In summary, synthetic materials for repairing peripheral nerve injuries are typically characterized by their degradability, high external strength, slow degradation rates, and cell growth-promoting properties in the inner layer.

In recent years, GelMA has gained widespread application in tissue engineering fields such as bone, muscle, cartilage, and heart due to its excellent biocompatibility and ease of modification [9]. The utilization of GelMA for repairing peripheral nerve injuries is still in its nascent stages. Dursun, U.T., Soucy, J.R., et al. [10,39] developed GelMA-based composite hydrogels, which were found to enhance the adhesion and proliferation of Schwann cells and promote the directional distribution of PC12 (a nerve cell line). Zhuang, H. et al. [40] encapsulated glial cell line-derived neurotrophic factor (GDNF) within GelMA microspheres and injected it into a nerve sheath constructed from a commercial collagen membrane (Bio-Gide(^®^)) to repair sciatic nerve defects in rats. Hu, Y et al. [41] and Gong, H et al. [42] utilized 3D printing technology and cryopolymerization methods to fabricate GelMA nerve conduits that promoted the adhesion, proliferation, and expression of neural-related genes in adipose-derived stem cells. Although the 3D-printed GelMA nerve conduits effectively facilitated the repair of 10 mm sciatic nerve defects in rats, their poor mechanical properties and rapid degradation led to gradual disintegration and collapse during experiments, preventing the formation of a stable tubular structure and thus failing to meet the requirements for peripheral nerve regeneration.

In this study, to address the issues of poor mechanical properties and rapid degradation rate of GelMA, we adopted a composite material strategy by incorporating PCL with GelMA. Zhao et al. [15] fabricated GelMA-PCL composite nanofibers via electrospinning technology, which exhibited a cross-linked and linear arrangement structure. Importantly, these composite nanofibers were shown to promote the remodeling of vascular endothelial cells. Therefore, we chose to combine PCL with GelMA to enhance its mechanical strength and slow down its degradation rate. However, the cross-linked and linear arrangement of GelMA and PCL alone cannot fully meet the requirements for peripheral nerve repair, such as adequate mechanical strength, hydrophilicity, and controlled degradability, necessitating further modification.

In general, the inherent hydrophilic and hydrophobic properties of GelMA and PCL, respectively, hinder their combination into a homogeneous solution, leading to significant phase separation (Figure 1C). In this study, after mixing PCL and GelMA in equal proportions, a stable and uniform solution was achieved by adjusting the pH, indicating that the phase separation between GelMA and PCL was mitigated. The primary component of GelMA is gelatin, a protein rich in amino acid groups. In trifluoroethanol solution, the hydrogen bonds within gelatin molecules are disrupted, shifting the pH far from its isoelectric point, thereby adopting a linear structure and rendering the solution transparent. However, as PCL is hydrophobic, adding the GelM–trifluoroethanol solution causes GelMA to adopt a hydrophobic state, with its hydrophilic groups becoming encapsulated internally while hydrophobic groups are exposed externally, ultimately leading to gelatin aggregation and precipitation. After pH adjustment, GelMA molecules carry like charges, repelling each other and reverting to their original linear structure. This allows for weak interactions between the hydrophobic groups of GelMA and PCL, thus overcoming phase separation and maintaining solution transparency (Figure 1C). Clearly, achieving a uniform and stable solution is crucial for successful electrospinning. During electrospinning, the pH of the mixture reverts to its initial state due to solvent evaporation post-extrusion, causing phase separation between GelMA and PCL to reoccur. Additionally, upon reoccurrence of phase separation, GelMA undergoes polymerization under ultraviolet light, resulting in spontaneous encapsulation on the PCL surface and formation of a core–sheath-like structure.

Compared with pure PCL nanofibers, the core–sheath structure significantly reduces both the diameter and diameter distribution of the nanofibers. Moreover, a smaller nanofiber diameter results in a larger specific surface area, which more closely mimics the natural ECM morphology. The small pores (<10 μm) between fibers on the tube wall facilitate material exchange between intraluminal metabolites and extraluminal nutrients when used in animal models [43]. Additionally, the appropriate nanofiber diameter enhances the proliferation and differentiation potential of stem cells [44]. Furthermore, the core–sheath structure significantly enhances the hydrophilicity of the nanofibers, achieving complete hydrophilicity—a marked improvement over previous findings reported in the literature [15]. Enhanced hydrophilicity is beneficial for cell adhesion and extension.

There are MMP-sensitive sites in GelMA that react with collagenase to induce degradation [9,45], while PCL contains hydrolytically unstable aliphatic ester bonds that also react with collagenase to cause degradation [12]. In vitro degradation tests demonstrated that the degradation rate of GelMA/PCL nanofibers in the core–sheath structure was rapid (exceeding 40%) within the first 7 days, primarily due to the fast degradation rate of GelMA. When exposed on the surface of composite nanofibers, GelMA initiates degradation first. Over the subsequent 21 days, the degradation rate slowed significantly, as most of the GelMA had degraded within the initial 7 days, exposing PCL, which degrades at a much slower rate. GelMA incorporates RGD domains and MMP-sensitive degradation sites that promote cell adhesion and remodeling [9,46]. The core–sheath structure allows GelMA to directly interact with cells upon exposure, influencing cellular behavior, and its degradation products can affect neuron-related cells. Following the rapid degradation of GelMA, the slowly degrading PCL maintains the hollow tubular structure of the nerve cannula without collapse, providing space for nerve regeneration. Therefore, the core–sheath structure of GelMA/PCL nanofibers not only fulfills the biocompatibility requirements for peripheral nerve regeneration but also meets the temporal demands for nerve regeneration. This study did not include in vivo degradation assessment, representing a recognized methodological limitation inherent to the current experimental scope.

In addition to an appropriate degradation profile, superior mechanical properties are critical determinants of peripheral nerve conduit performance. An excessively rigid conduit may provoke chronic compressive neuropathy during regeneration, whereas insufficient stiffness compromises structural stability—leading to luminal collapse and impaired axonal guidance. Although poly(ε-caprolactone) (PCL) exhibits lower resistance to elastic deformation than gelatin methacryloyl (GelMA), their synergistic integration significantly enhances the elastic modulus of the resulting composite nanofibers. This improvement enables the conduit to sustain physiological mechanical loads without irreversible deformation or loss of architectural fidelity. Consequently, an optimal conduit design must reconcile two biomechanical imperatives: sufficient stiffness to maintain patency and provide regenerative guidance, and tailored elasticity to match the viscoelastic behavior—including elastic modulus and strain recovery—of native peripheral nerve tissue. Our results demonstrate that the electrospun nerve conduit is capable of maintaining its hollow tubular architecture and preserving its three-dimensional structural integrity for up to 12 weeks following transplantation in rats.

In vitro studies are widely recognized as the primary method for evaluating the biocompatibility of biomaterials. Consequently, it is essential to assess the in vitro biocompatibility of GelMA/PCL nanofibers prior to conducting in vivo experiments. In this study, scanning electron microscopy (SEM) demonstrated that Schwann cells adhered efficiently to both core–sheath-structured GelMA/PCL nanofibers and pure PCL nanofibers, with no statistically significant differences detected between the two groups, a finding consistent with previous literature [15,16,17]. Quantitative analysis of Schwann cell growth and proliferation co-cultured with the nanofibers at two distinct time points demonstrated that both the core–sheath-structured GelMA/PCL nanofibers and pure PCL nanofibers exhibited excellent biocompatibility, with no significant differences noted between them.

Clements, M.P. et al. [47] reported that the microenvironment following peripheral nerve injury can induce Schwann cells to dedifferentiate and reprogram into mesenchymal stem cell-like states. Masaki, T. et al. [48] demonstrated that stimulation of mature Schwann cells with Mycobacterium leprae can also trigger their reprogramming into stem cell-like states. Zhao, Q. et al. [15] further noted that GelMA/PCL composite scaffolds promote vascular endothelial cell remodeling, highlighting the potential of external stimuli in altering cell fate, including regression to a stem cell state. A notable characteristic of Schwann cells is their ability to reverse developmental processes, transitioning from mature to immature states upon nerve damage or external stimulation [47,49,50]. Additionally, Schwann cells at different stages express distinct markers, allowing for the assessment of their differentiation status through marker detection. Previous studies have shown that genes such as p75 and Krox24 are activated and up-regulated in dedifferentiated Schwann cells [51,52]. It has been reported that Sox2 and c-Jun are co-expressed in dedifferentiated Schwann cells following peripheral nerve injury, with their expression levels significantly up-regulated. The increased expression of Sox2 inhibits the expression of Krox-20 and cAMP. The balance between the transcription factors c-Jun (promoting dedifferentiation) and Krox-20 (promoting differentiation) is a critical regulator of Schwann cell differentiation/dedifferentiation (myelination/demyelination) states [47,53]. Additionally, Sox2 is a characteristic gene of stem cells [54]. Our results demonstrate that the GelMA/PCL nanofiber scaffold significantly up-regulated the expression of Krox24, p75, and Sox2 genes in Schwann cells, thereby activating and highly expressing dedifferentiation-related genes. However, it is important to note that our study is preliminary, and further experimental validation is required to confirm whether true dedifferentiation of Schwann cells occurs. Cyclin D1, which promotes cell proliferation, is expressed in both differentiated and dedifferentiated Schwann cells [51,55]. The GelMA/PCL nanofiber scaffold significantly up-regulated Cyclin D1 gene expression in Schwann cells, promoting their proliferation, consistent with CCK8 assay results.

The rat model of sciatic nerve defect is a widely accepted preclinical model for evaluating the efficacy of nerve guidance conduits. In this study, the model was utilized to assess the performance of GelMA/PCL nanofiber scaffold-based neural conduits in repairing a 10 mm sciatic nerve defect in rats. Comprehensive assessments, including electrophysiological analysis, functional behavioral testing, and histomorphological examination, were carried out to evaluate axonal regeneration and functional recovery outcomes. This study did not incorporate key control groups—specifically, the surgical control group (undergoing nerve transection only, without repair) and the empty conduit control group (implanted with an acellular, matrix-free conduit)—a methodological constraint explicitly acknowledged in the Discussion section.

The results of gait analysis not only reflect the functional recovery following nerve defect repair but also provide an effective means for assessing mechanically induced pain. It was observed that following the repair of nerve defects in rats using a core–sheath-structured GelMA/PCL nanofiber scaffold nerve conduit, the mechanical pain response, foot condition, and motor coordination were comparable to those achieved through autologous nerve transplantation. Electrophysiological testing represents another widely utilized approach for evaluating nerve repair outcomes, wherein CMAP serves as an indirect indicator of both the quantity of regenerated nerve fibers and the extent of neuromuscular reinnervation. Furthermore, nerve fiber diameter, axon diameter, and myelin sheath thickness collectively influence MNCV. In this study, both CMAP and MNCV values in the GelMA/PCL group demonstrated significant enhancement compared with the PCL group, with outcomes closely approaching those observed in the autologous nerve transplantation group.

Neurohistological examination allows for direct visualization of regenerated nerves within the canal. Following immunofluorescence staining of nerve tissue, it is evident that the GelMA/PCL group exhibits a substantial number of regenerated nerve fibers with nuclei aligned in an orderly manner, indicating active cellular behavior at the site of nerve regeneration. Additionally, Kim et al. [27] demonstrated that when using directionally aligned polyacrylonitrile–methyl acrylate nanofiber nerve conduits to repair nerve defects in rats, the regenerated nerves contained a significant number of vascular endothelial cells. Consistent with these findings, our study also identified vascular endothelial cells within the nerve conduit. Furthermore, Cattin et al. [56] observed in their investigation of peripheral nerve mechanisms under hypoxic conditions that macrophages induce vascular polarization to form a “track” and recruit Schwann cells to aggregate, guiding Schwann cell migration towards the distal end of the nerve along this “track”. Our research similarly found that Schwann cells and vascular endothelial cells coexist, although the specific relationship between these two cell types requires further investigation.

Peripheral nerve fibers terminate in various tissues or organs, forming a diverse array of nerve ending structures. The axons of motor neurons innervate muscle tissue, thereby controlling its contractile activity. Motor nerve endings and their associated muscle fibers constitute effector units known as neuromuscular junctions (motor endplates). The neuromuscular junction is a highly specialized structure composed of numerous cholinergic synapses that regulate muscle function. Following nerve injury, the corresponding neuromuscular junction undergoes structural degeneration and atrophy. Successful nerve regeneration leads to the re-establishment of synaptic connections between motor nerve endings and muscle fibers. Terminal Schwann cells actively participate in the formation of neuromuscular junctions and the growth of nerve branches, guiding regenerating axons toward muscle fibers and promoting the remodeling of neuromuscular junctions. Our results demonstrate that, in the GelMA/PCL group, regenerated axons and Schwann cells effectively re-innervated muscle fibers, restoring both the structural integrity and partial functional capacity of the neuromuscular junction, as evidenced by the presence of synaptic vesicles, with outcomes comparable to those achieved through autologous nerve graft repair.

Following nerve disconnection, the corresponding skeletal muscle undergoes denervation-induced atrophy and degeneration. However, if nerve regeneration and successful reinnervation of the target organ occur, these pathological changes can be partially prevented and reversed. Our study demonstrated that the GelMA/PCL group not only facilitated structural reconstruction of the neuromuscular junction but also restored certain functional capabilities, resulting in a statistically significant improvement in the wet-weight ratio of both the tibialis anterior and gastrocnemius muscles.

## 5. Conclusions

In summary, this study demonstrates, for the first time, a straightforward yet adaptable method for fabricating unique core–sheath GelMA/PCL nanofibers. The phase separation and polymerization that occur during the electrospinning process lead to the formation of a distinct core–sheath structure. This structural configuration exerts a significant influence on the physicochemical and biological properties of the nanofibers, including wettability, degradation rate, mechanical characteristics, and cellular interactions. Notably, regenerated axons were able to bridge 10 mm nerve gaps using core–sheath GelMA/PCL nanofiber nerve guidance conduits (NCGs), successfully reinnervate target muscles and reconstruct functional neuromuscular junctions. These findings suggest that core–sheath GelMA/PCL nanofiber NCGs hold considerable potential as an alternative to autografts, which are currently the clinical gold standard for repairing long peripheral nerve defects.

## Data Availability

The original contributions presented in this study are included in the article/Supplementary Materials. Further inquiries can be directed to the corresponding authors.

## Supplementary Materials

The following supporting information can be downloaded at: https://www.mdpi.com/article/10.3390/polym18101241/s1. Figure S1. The diameter distribution of pure PCL nanofibers and core-sheath GelMA/PCL nanofibers. Figure S2. Confocal microscopy images of FITC-labeled pure PCL nanofibers and core-sheath GelMA/PCL nanofibers. Figure S3. The surface wettability of pure PCL nanofibers and core-sheath GelMA/PCL nanofibers. Figure S4. Mechanical properties of pure PCL nanofibers and core-sheath GelMA/PCL nanofibers. Figure S5. Walking track analysis. Figure S6. Electrophysiological and behavioral outcomes. Figure S7. Immunofluorescent analysis of nerve regeneration. Figure S8. Analysis of the myelinated nerve fibers based on TEM images. Figure S9. Morphological images of muscles and muscle wet weight ratios. Table S1. The primers used for RT-PCR were shown below. Video S1: Video recording of contact angle measurements performed on pure PCL nanofibrous membranes and core–sheath GelMA/PCL nanofibrous membranes. Video S2: Walking track analysis. Form S1: ARRIVE Checklist.
