# Supplementary material for: Biomimetic Core–Sheath GelMA/PCL Nanofibers for Enhanced Peripheral Nerve Regeneration
Source: Polymers (Basel). 2026 May 19;18(10):1241. doi: 10.3390/polym18101241 (PMC13211255; doi:10.3390/polym18101241)
Supplement: Supplementary file 1 [file polymers-18-01241-s001.zip › Fig S1-S9.Tab S1.pdf]

Biomimetic Core-Sheath GelMA/PCL Nanofibers for Enhanced Peripheral Nerve Regeneration

Xingxing Fang[1], Haichang Guo[3], Fei Yu[4], Wei Zhang[1], Qicheng Li[2], Shulin Bai[5]*, Peixun Zhang[2]*

1. Department of Spine Surgery, The Third Affiliated Hospital of Sun Yat-sen University, Guangzhou, Guangdong Province, 510630, China

2. Department of Orthopedics and Trauma, Peking University People's Hospital, Beijing, 100044, China

3. Department of Electronic Engineering, The Chinese university of Hong Kong, Hongkong, 999077, China

4. Department of Spine Surgery, Shenzhen Second People's Hospital, The First Affiliated Hospital of Shenzhen University, Shenzhen, 518035, China

5. School of Materials Science and Engineering, Peking University, Beijing, 100871, China

*Corresponding authors: Shulin Bai: slbai@pku.edu.cn, Peixun Zhang: zhangpeixun@bjmu.edu.cn

Supplementary Data

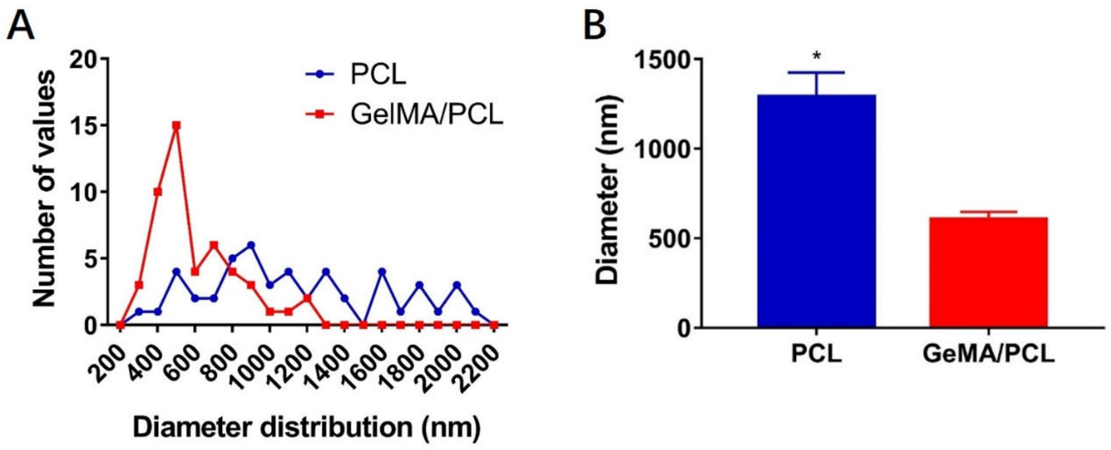

Figure S1. The diameter distribution of pure PCL nanofibers and core-sheath GelMA/PCL nanofibers, as measured on 50 randomly selected fibers.

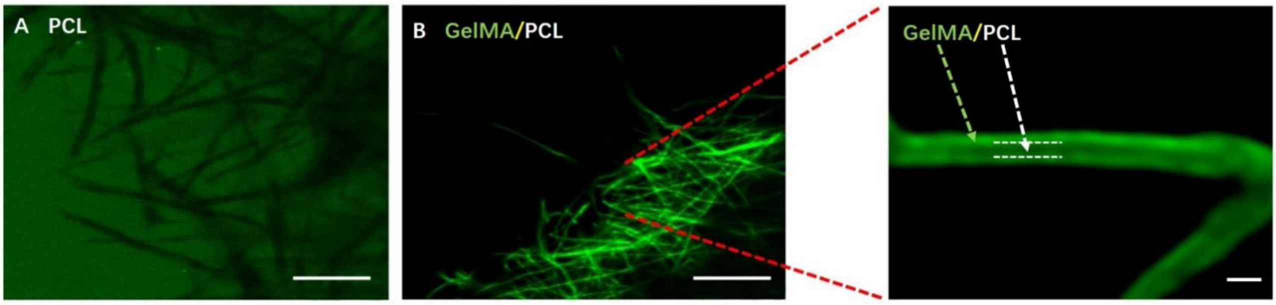

Figure S2. A) and C) Confocal microscopy images of FITC-labeled pure PCL nanofibers and core-sheath GelMA/PCL nanofibers, respectively. Scale bar = 10 μm. B) is a magnified view of image A). Scale bar = 500 nm.

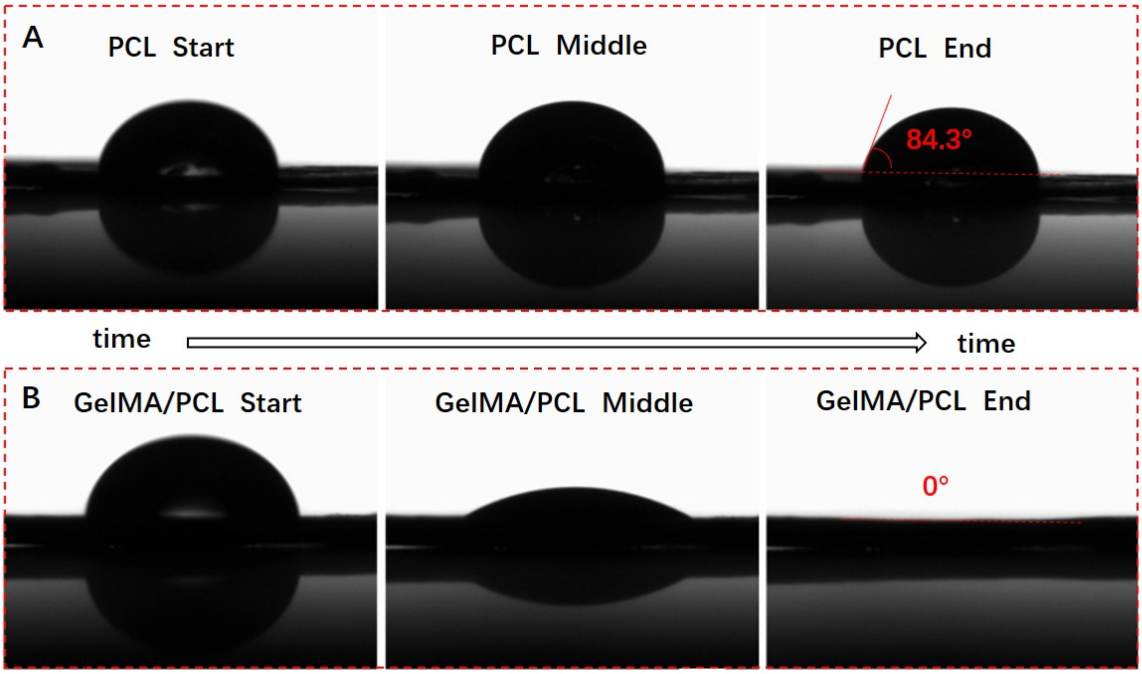

Figure S3. A) and B) The surface wettability of pure PCL nanofibers and core-sheath GelMA/PCL nanofibers. Over time, the water contact angle of the core-sheath GelMA/PCL nanofibers decreased to 0°, in contrast to 84.3° for PCL. Experimental procedure is documented in the Supplementary Video.

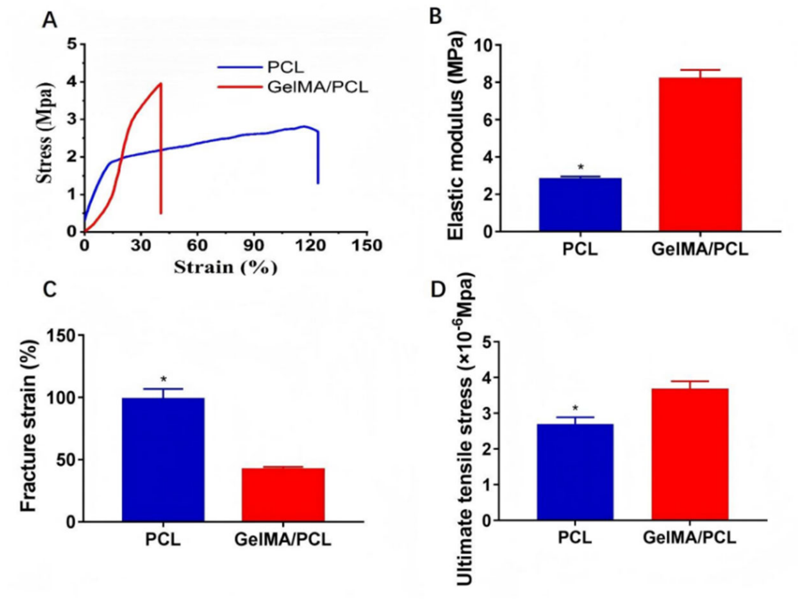

Figure S4. A–D) Mechanical properties of pure PCL nanofibers and core-sheath GelMA/PCL nanofibers: tensile strength (A), elastic modulus (B), fracture strain (C), and ultimate tensile stress (D). *P < 0.05. Error bars represent the standard error of the mean (s.e.m.).

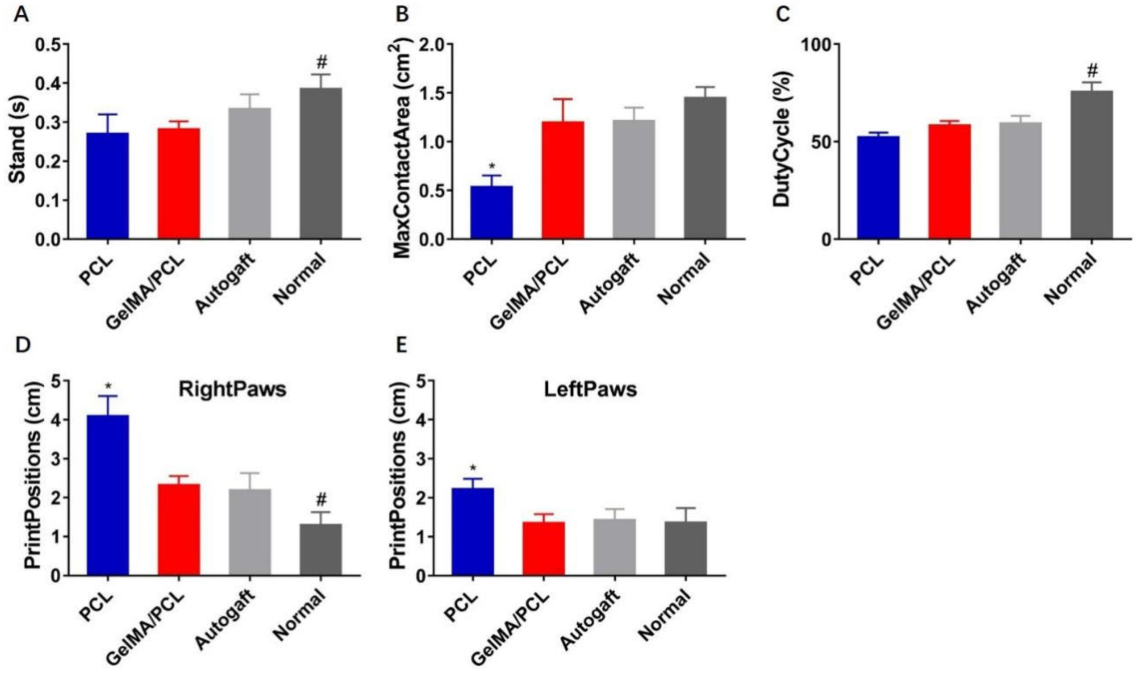

Figure S5. Walking track analysis. A–E) Gait parameters of the PCL, GelMA/PCL, and Autograft groups, respectively: stance duration (A), maximum contact area (B), duty cycle (C), right paw print position (D), and left paw print position

(E). *P < 0.05, #P < 0.05. Error bars represent the standard error of the mean (s.e.m.). n=6.

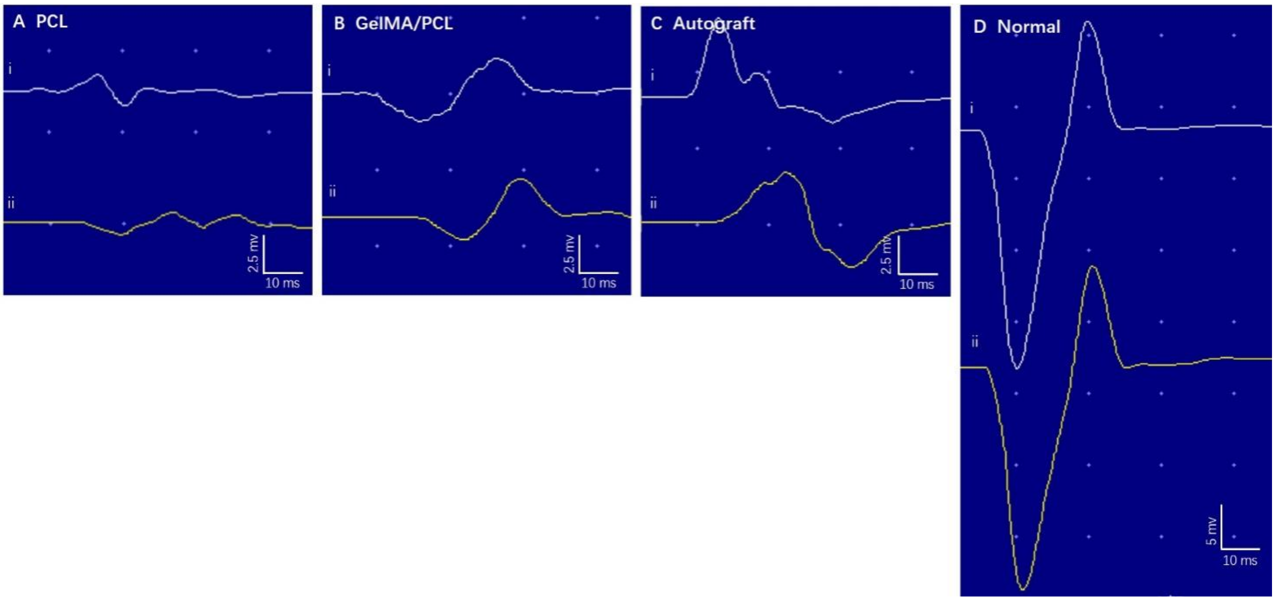

Figure S6. Electrophysiological and behavioral outcomes. A–D) Compound muscle action potentials (CMAPs) recorded from the PCL, GelMA/PCL, Autograft, and Normal groups following electrical stimulation at the distal (i) and proximal (ii) sites of the sciatic nerve.

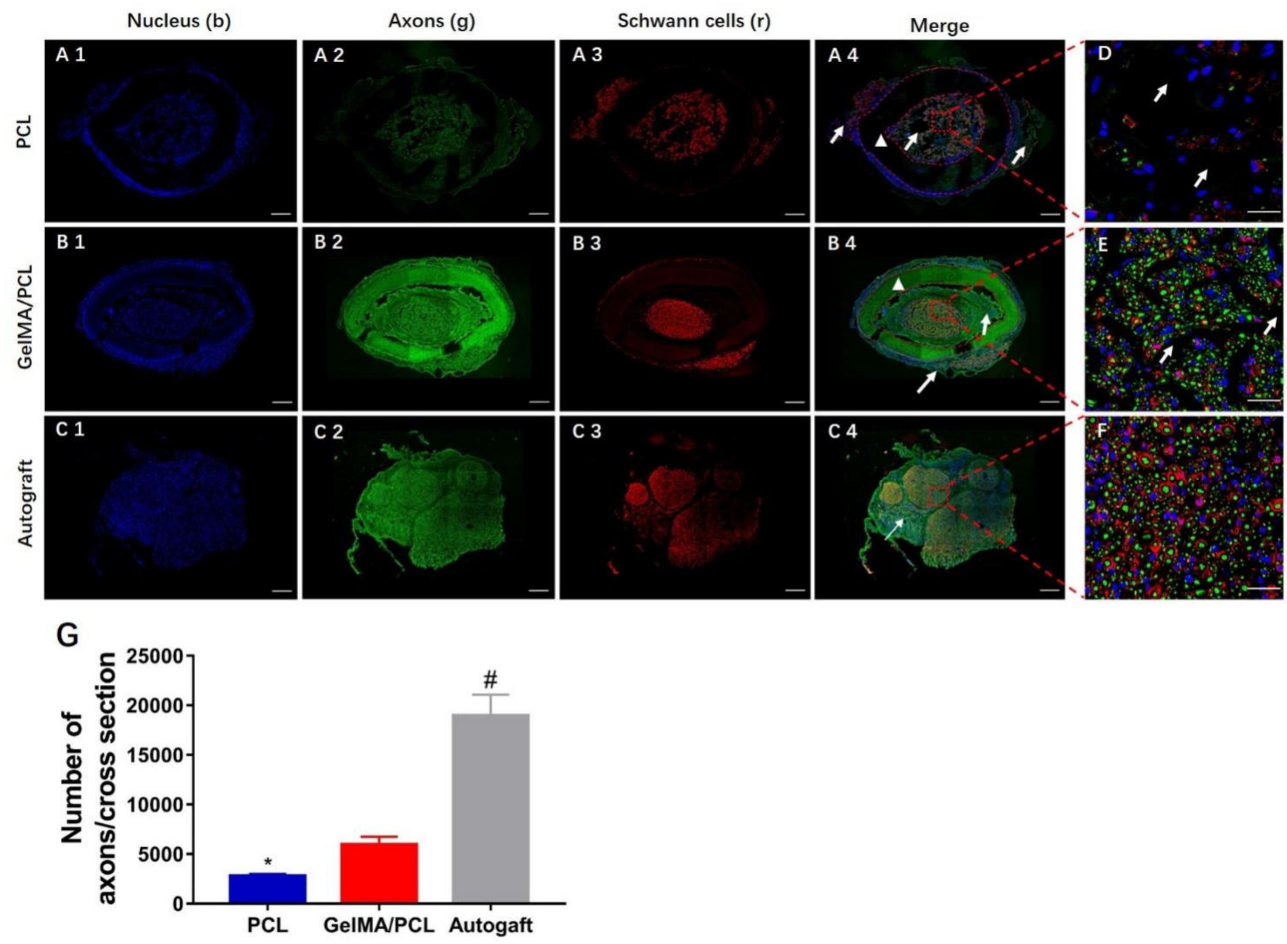

Figure S7. Immunofluorescent analysis of nerve regeneration (longitudinal section, mid-implantation region). A) PCL group; B) GelMA/PCL group; C) Autograft group. Double labeling shows regenerating axons (green) and Schwann cells (red). White triangles indicate pure PCL nanofiber nerve guidance conduits or core-sheath GelMA/PCL nanofiber nerve guidance conduits. White arrows point to axon/Schwann cell-depleted regions. Scale bar = 25 μm. A1–A3, B1–B3, and C1–C3) magnified views of A, B, and C, respectively. Scale bar = 250 μm. G) Immunofluorescent analysis of nerve regeneration (transverse cross-section, mid-implantation region). Quantitative comparison of the average number of axons per cross-section among the PCL, GelMA/PCL, and Autograft groups. $*P < 0.05$, $\#P < 0.05$. Error bars represent the standard error of the mean (s.e.m.). n=3 (The quantity was reduced by half because it was used for the immunohistochemical experiment on the nerve cross-section.).

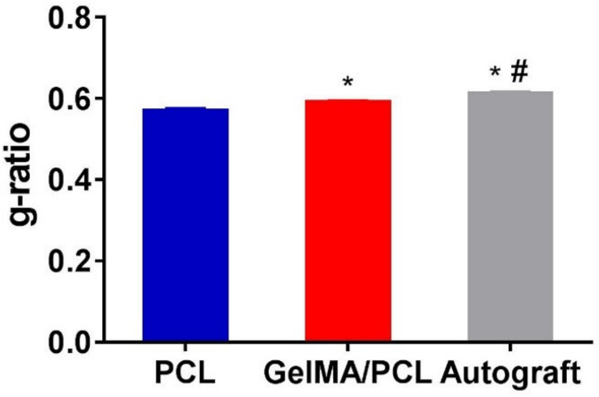

Figure S8. Analysis of the myelinated nerve fibers based on TEM images, n = 6, *: P < 0.05, compared with the PCL group; #: P < 0.05, compared with the GelMA/PCL group

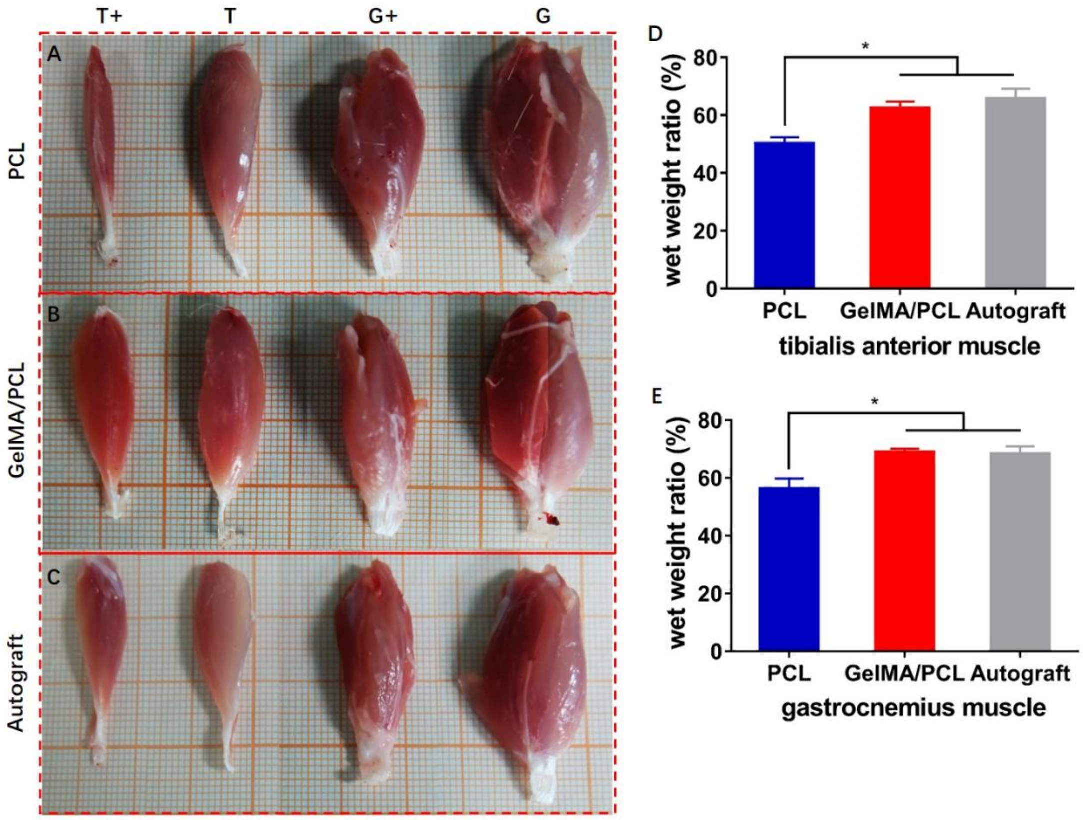

Figure S9. Morphological images of muscles and muscle wet weight ratios. Representative morphological images of the tibialis anterior muscles from the PCL group (A), GelMA/PCL group (B), and Autograft group (C). Experimental side tibialis anterior muscles (T+), contralateral side tibialis anterior muscles (T), experimental side gastrocnemius muscles (G+), and contralateral side gastrocnemius muscles (G). D) and E) Ratios of tibialis anterior muscle wet weight and gastrocnemius muscle wet weight. *P < 0.05, #P < 0.05. Error bars represent the standard error of the mean (s.e.m.). n=6.

Table S1. The primers used for RT-PCR were shown below.

Gene	Primers
Krox24	Forward primer 5'-AGAGGGCAGCGGCGGCAATAA-3'
	Reverse primer 5'-GGAGGCAGAGGAAGACGATGAAGCAG-3'
Cyclin D1	Forward primer 5'-CGAGGAGCAGAAGTGCGAAGAGG-3'
	Reverse primer 5'-GGGCGGATAGAGTTGTCAGTGTAGATG-3'
p75	Forward primer 5'-AGATGTGCCTATGGCTACTACCAGGACGAG-3'
	Reverse primer 5'-CCAAGATGGAGCAATAGACAGGAATGAGG-3'
Sox2	Forward primer 5'-CGCACATGAACGGCTGGAGCA -3'
	Reverse primer 5'-GCCCTGGAGTGGGAGGAAGAGGTAA-3'
GAPDH	Forward primer 5'-CGGCAAGTTCAACGGCACAGTCA-3'
	Reverse primer 5'-CACGCCACAGCTTTCCAGAGGG-3'
